# The Mother Centriole Plays an Instructive Role in Defining Cell Geometry 

**DOI:** 10.1371/journal.pbio.0050149

**Published:** 2007-05-22

**Authors:** Jessica L Feldman, Stefan Geimer, Wallace F Marshall

**Affiliations:** 1 Department of Biochemistry and Biophysics, University of California San Francisco, San Francisco, California, United States of America; 2 Biologie/Elektronenmikroskopie NW I/B 1, Universität Bayreuth, Bayreuth, Germany; Osaka University, Japan

## Abstract

Centriole positioning is a key step in establishment and propagation of cell geometry, but the mechanism of this positioning is unknown. The ability of pre-existing centrioles to induce formation of new centrioles at a defined angle relative to themselves suggests they may have the capacity to transmit spatial information to their daughters. Using three-dimensional computer-aided analysis of cell morphology in *Chlamydomonas,* we identify six genes required for centriole positioning relative to overall cell polarity, four of which have known sequences. We show that the distal portion of the centriole is critical for positioning, and that the centriole positions the nucleus rather than vice versa. We obtain evidence that the daughter centriole is unable to respond to normal positioning cues and relies on the mother for positional information. Our results represent a clear example of “cytotaxis” as defined by Sonneborn, and suggest that centrioles can play a key function in propagation of cellular geometry from one generation to the next. The genes documented here that are required for proper centriole positioning may represent a new class of ciliary disease genes, defects in which would be expected to cause disorganized ciliary position and impaired function.

## Author Summary

Cells are not just homogenous bags of enzymes, but instead have a precise and complex internal architecture. However, the mechanisms that define this architecture remain unclear. How do different organelles find their proper location within the cell? We have begun to address this question for one particular organelle, the centriole, using a genetic approach. Our approach relies on the fact that centrioles are required for the assembly of cilia and flagella, which are used for swimming. We studied the unicellular green alga *Chlamydomonas,* which use flagella to swim towards a light source. We screened for mutants that could not swim towards light, and found a set of mutants in which the centrioles and flagella are displaced from their normal location within the cell. Using these mutants, we have obtained evidence that centrioles play a role in positioning other structures within the cell, such as the nucleus. We also found that in these cells, which contain two centrioles differing in age, the older centriole plays a role in positioning the newer centriole, suggesting that cells may have a way to propagate spatial patterns from one generation to the next.

## Abstract 3

Six genes required for centriole positioning are identified in*Chlamydomonas.* Analysis of cells with mutations in these genes reveals a crucial role for the centriole in positioning other organelles.

## Introduction

A fundamental question in cell biology is how cell geometry is established and maintained [1–4]. Cell geometry refers to the characteristic positioning of organelles within the cell body in order for a cell to be able to carry out its specified function. Despite the importance of cell geometry in tissue organization and cell function, the mechanis-tic origins of cell geometry remain a mystery. Further compounding the mystery is the fact that, as demonstrated by the classic experiments of Beisson and Sonneborn [5], cell organization can be propagated through cell division, alleviating the need for cells to re-establish their infrastructure after each round of mitosis, and potentially allowing a coherent organization to be maintained across developing tissue during proliferative growth. Many organelles take part in this elaborate cellular patterning. One organelle that is often found in specific subcellular locations is the centriole.

Centrioles are non–membrane-bound organelles composed of nine triplet microtubule blades arranged around a central cartwheel structure. Centrioles are found as a pair, composed of a mother and a daughter, which is duplicated during each cell cycle. Mother centrioles are so-called because they were assembled in a previous cell cycle to the daughter centriole. Mother centrioles have unique ultrastructural modifications [6] and are decorated with a number of molecules not found on daughter centrioles.

Centrioles have two main functions in the cell. First, centrioles together with pericentriolar material comprise the centrosome, the major microtubule-organizing center of the cell. Indeed, centrioles are the highly stable, core nucleating centers for the centrosome, providing it with persisting structural integrity [7] and attaching it to cytoplasmic microtubules during G1 [8]. Second, centrioles serve as basal bodies to nucleate the assembly of cilia. In order to carry out these functions in the cell, centrioles often need to be specifically localized.

Although originally named for their centralized location, centrioles are repositioned to more peripheral sites during cell-state transitions such as wound healing, cell migration, and cell growth [9–11]. The importance of centriole positioning for development and physiology is perhaps most clearly illustrated in situations involving cilia, which are assembled from centrioles. The problem of ciliary positioning is 2-fold. First, centrioles must migrate to the proper region on the cell surface where they will dock and assemble cilia. Second, once centrioles reach the cell surface, they must become properly oriented so as to create a proper directional stroke in the case of motile cilia, or so they are oriented to participate in signaling as in the case of a primary cilium. Perturbation in either step of ciliary positioning has severely deleterious effects in humans [12]. For example, inability of centrioles to properly migrate prior to ciliary assembly has recently been linked to Meckel-Gruber syndrome [13]. Additionally, proper orientation of cilia via centriole positioning towards the posterior of embryonic node cells is critical for establishing left–right asymmetry during mammalian development [14]. Centrioles must also be properly positioned when they serve as basal bodies in multiciliated cells such as in the tracheal epithelium. Centriole orientation, and the resulting proper alignment of respiratory cilia, is required for effective mucus clearing in the airway [15]. In all cases in which cilia act either to drive fluid flow or act as sensors, it is important that they be placed on the appropriate region of the cell surface; for example, in cells lining a duct, the cilia would have to face the lumen of the duct, which requires specific positioning of centrioles on a limited patch of cell surface.

It is clear that centriole positioning is critical in many aspects of cell behavior, especially in placing a cilium that will interact with the extracellular environment. Centriole position may also serve a function in intracellular events. As centrioles are anchored to the cytoskeleton during G1, they may act as a set of stable “handles” by which the centrosome can be repositioned to orient the cytoskeleton, cilia, and perhaps, other cellular structures as well. Moreover, the process of centriole duplication provides an ideal mechanism to transmit cell geometry across generations. Although both planar cell polarity [16,17] and apical/basal cues [18,19] can influence centriole position, the mechanism by which centrioles are positioned, and the degree to which their positioning is self-propagating, is currently unknown.

**Figure 1:**
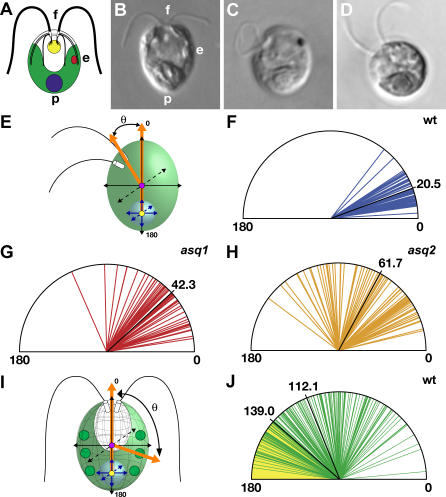
Identification and Quantification of Defects in *asq* Mutants (A) *Chlamydomonas* cell geometry. Flagella (f) extend from the centrioles (white), which are located apically and are attached to the nucleus (yellow) by centrin-containing fibers. The pyrenoid (p; blue), a starch-containing structure, is located basally and is embedded in a cup-like mass of chloroplast (green). The eyespot (e; red), the light-sensing organelle, is located laterally at a reproducible angle relative to the centrioles. (B) DIC image of a wt *Chlamydomonas* cell. The pyrenoid (p), eyespot (e), and flagella (f) are indicated. All DIC images are sections through full 3D datasets. (C) In *asq1* cells, mother–daughter centriole pairs are randomly localized on the cell surface. (D) In *asq2* cells, centrioles are independently positioned on the cell surface and no longer found in pairs. (E) Defining θ_centriole_. A 3D vector reflecting the long axis of the cell is drawn from the center of mass (yellow circle) of the pyrenoid (blue) to the cellular center of mass (purple circle). θ_centriole_ is the angle between the vector defining the long axis of the cell and the vector from the cellular center of mass to each centriole (white). All angle measurements are made in three dimensions using 3D image datasets. (F) The mean θ_centriole_ (black line) for wt cells is 20.5 ± 9.0° (*n* = 62), (G) θ_centriole_ increases to 42.3 ± 21.3° for *asq1* cells (*n* = 54). Angles are biased toward the top half of the cell, presumably because the lower half of the cell is occluded by the chloroplast, which is localized in the basal half of the cell body (see [J]) and closely apposed to the plasma membrane, thus reducing access of basal bodies to the cell surface. (H) θ_centriole_ increases to 61.7 ± 32.3° for *asq2* cells (*n* = 71). (I) Defining θ_chloroplast_. The cell center–pyrenoid axis is defined as described in (E). θ_chloroplast_ is defined as the angle between the vector defining the long axis of the cell and the vector from the cellular center of mass to each plastid genome (large green circle). (J) The θ_chloroplast_ for wt cells is shown in green (mean = 112.1 ± 36.0°, *n* = 181). Each line represents the position of one plastid genome. The yellow-shaded area represents the area of the cell occupied by the pyrenoid. The non-180° edge of this shaded region indicates the mean position of the pyrenoid boundary (mean = 139.0 ± 14.4°, *n* = 90).

The unicellular alga Chlamydomonas reinhardtii provides an ideal genetic system in which to study centriole positioning. Each pair of centrioles, composed of a mother and a daughter, must relocate from the apical cell surface to the spindle poles during mitosis. After division, centrioles return to the apical pole where they nucleate the assembly of two cilia (called flagella in this organism). *Chlamydomonas* centrioles and cilia are structurally similar to those of vertebrates, with the vast majority of centriolar and ciliary proteins conserved between humans and *Chlamydomonas*. *Chlamydomonas* cells also have reproducible chiral cell geometry with many characteristically positioned structures [20] (illustrated in Figure 1A and 1B), facilitating quantification of geometric relationships within the cell. Given the importance of cilia positioning in animal tissues, and the high conservation of the ciliary apparatus components between *Chlamydomonas* and animals, we feel that this unicellular alga is an excellent gene-discovery platform for analyzing cilia-placement mechanisms that may turn out to be important in human ciliary diseases.

Using *Chlamydomonas* cells, we identified mutants with defects in centriole positioning. Combining genetic analysis, three-dimensional (3D) imaging, and a novel algorithm for quantifying cellular geometry, we demonstrate that the mother centriole guides the daughter centriole to the proper subcellular location. Specifically, in mutants in which mother and daughter centrioles are separated, only mother centrioles localize properly. We further show that in mutants in which the centrioles are detached from the nucleus, the nucleus becomes randomly positioned, whereas the mother centrioles retain correct positioning, indicating that normally, the mother centriole plays a role in properly positioning the nucleus and not vice versa. These data indicate that the mother centriole may act as a node to coordinate the positioning of many subcellular structures.

## Results

### Phototaxis Screen Uncovers Mutants with Defects in Centriole Positioning

To initiate a genetic analysis of the mechanism of centriole positioning and its impact on cell geometry, we began with a screen based on *Chlamydomonas* phototaxis*. Chlamydomonas* cells phototax using a light-sensing organelle called the eyespot. Cells rotate while swimming, sweeping out a 360° path, looking for light. When the eyespot detects light, it signals to the flagella via calcium signaling, inducing the cell to turn towards the light [21]. We predicted that cells with aberrantly placed centrioles, and therefore, aberrantly placed flagella, would lack the geometric relationship between the eyespot and the flagella that is required for phototaxis, and would be revealed in a screen for phototaxis defects. We screened 10,000 insertionally mutagenized lines for defects in phototaxis using an assay similar to previously described techniques [22–24]. Phototaxis-defective lines were visually rescreened by differential interference contrast (DIC) microscopy to identify mutants with defective cell morphology. Screen details are listed in Figure S1.

Centriole positioning mutants were identified as those whose flagella are displaced from the apical pole of the cell (the usual position of centrioles in G1 in *Chlamydomonas*) and were verified using a 3D computer-aided image analysis strategy as follows. We defined the long axis of the cell using the center of mass of the pyrenoid (Figure 1E, yellow circle), a starch-storage structure that is located basally, and the cellular center of mass (Figure 1E, purple circle). We then marked the centrioles (Figure 1E, white cylinders), and using the long axis to construct a spherical coordinate system, we determined the angle by which each centriole was displaced off the long axis of the cell (θ_centriole_, Figure 1E). θ_centriole_ represents the zenith angle in a spherical coordinate system and is by definition between 0° and 180°. We were unable to measure the azimuth angle ϕ due to a lack of a visible reference point. We identified 13 mutants, which we termed *askew (asq),* in which centrioles are mispositioned as judged by θ_centriole_. For example, *asq1* cells have a mean θ_centriole_ of 42.3 ± 21.3° (Figure 1G, *n* = 54; all reported angles are the mean ± standard deviation). *asq2* cells have a mean θ_centriole_ of 61.7 ± 32.3° (Figure 1H, *n* = 71). These values differ significantly (one-tailed *t*-test, *asq1*: *p* < 5.4 e^−10^, *asq2*: *p* < 9.8 e^−17^) from wild-type (wt) cells, which have a mean θ_centriole_ of 20.5 ± 9.0° (Figure 1F, *n* = 62). The average angle in wt is non-zero because the two centrioles are on either side of the apical-most point, and hence displaced off the long axis.

**Figure 2:**
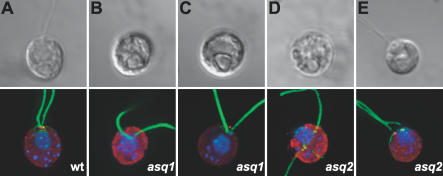
*asq* Mutants Can Be Divided into Two Classes Based on the Pairwise Distribution of Centrioles Images of fixed cells stained with DAPI and antibodies against centrin and acetylated tubulin (green) and Bld10p (red). DIC images are shown in the top panels, and fluorescence images of the same cells are shown below. All images are positioned so that the pyrenoid is located at the bottom. (A) wt cells have two centrioles located together at the apical side of the cell. One pro-centriole can be seen in the foreground of this image (red, marked by antibodies recognizing Bld10p). The other pro-centriole is occluded from view by the centriolar pair. (B and C) *asq1* cells have two centrioles that are positioned together at random locations on the cell surface. (D) In *asq2* cells, centrioles can be found at locations independent of one another. In this cell, both centrioles appear to be mispositioned. (E) In this *asq2* cell, one centriole along with its pro-centriole (marked by Bld10p staining in red) is found at the correct apical location. Another mispositioned centriole is found on the left, shifted off the long axis.

In *asq* cells, the angles tend to be restricted to the apical half of the cell due to the occlusion of the basal portion by other cellular structures. The basal portion and some of the apical portion of *Chlamydomonas* cells contain chloroplast. We measured the position of the chloroplast by using the same long-axis assignment described above. We then marked each plastid nucleoid (Figure 1I, green circles, visualized using DAPI, and Figure 2A, left) and determined the angle each nucleoid was displaced off the long axis of the cell. wt cells have a mean θ_chloroplast_ of 112.1 ± 36.0° (Figure 1J, *n* = 181). The pyrenoid center of mass is defined as 180° in all of our θ measurements because it is used as one of the points to define the long axis. The outer bounds of the pyrenoid span the basal part of the cell (Figure 1B). As was the case with the centrioles measurements, we calculate the zenith angle θ in standard spherical coordinates, which by convention can only vary between 0° and 180°. Thus, the bounds of the pyrenoid will both be less than 180°. The mean pyrenoid boundary in wt cells is 139.0 ± 14.4° (Figure 1J, yellow-shaded region, *n* = 90). The region of the cell that is occupied by the chloroplast and pyrenoid is thus complimentary to the region in which *asq* centrioles can be found, consistent with the notion that in *asq* mutants, centrioles are randomly distributed over the accessible part of the cell cortex.

*asq* mutants can be subdivided into two classes based on the pairwise association of centrioles. Normally, mother and daughter centrioles are held together by a system of connecting fibers. The *asq1* mutant represents a class of mutants (containing 9/13 *asq* mutants) in which mother and daughter centrioles are attached to each other as in wt, but are randomly localized together on the cell surface (Figures 1C, 2B, and 2C). The *asq2* mutant represents a second class (containing 4/13 *asq* mutants) in which the mother and daughter centrioles are independently positioned on the cell surface (Figures 1D, 2D, and 2E). In *asq2* cells, some centrioles appear at the correct apical location (Figures 2E and S5B), whereas other centrioles can occupy atypical positions (Figure 2D and 2E).

### Centriole Segregation Mutants Have Centriole Positioning Defects

**Figure 3:**
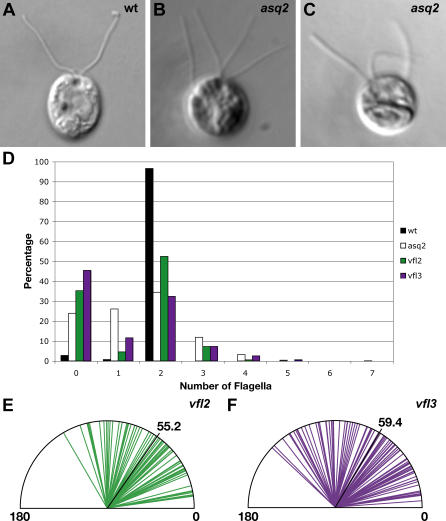
Mutants with Variable Numbers of Centrioles Also Have Centriole Positioning Defects (A) DIC image of a wt cell. wt cells have two flagella located at the apical side of the cells. (B and C) DIC images of *asq2* cells. *asq2* cells have variable numbers of centrioles and therefore make variable numbers of flagella. Some centrioles are randomly localized, whereas some are in the correct apical position. (D) Distribution of flagellar number in *asq2* cells is reminiscent of the *vfl* (variable flagellar number) phenotype. *asq2* cells (white) have a mean of 1.46 ± 1.1 flagella per cell (*n* = 1,274). This distribution is similar to that of *vfl2* cells (green; mean = 1.33 ± 1.05, *n* = 593) and *vfl3* cells (purple; mean = 1.12 ± 1.8, *n* = 466), but is in contrast to wt cells, which make two flagella (black; mean = 1.94 ± 0.34, *n* =1,005). (E) *vfl2* cells, previously identified as defective in centriole segregation, have a mean θ_centriole_ of 55.2 ± 28.8° (*n* = 64). (F) *vfl3* cells, defective in mother–daughter centriole cohesion, have a mean θ_centriole_ of 59.4 ± 35.2° (*n* = 90).

In addition to centriole positioning defects, *asq2* cells also have variable numbers of centrioles, and therefore make variable numbers of flagella (Figure 3B and 3C). In contrast to wt cells, which always have two flagella (Figure 3A and 3D, black bars), *asq2* cells can have from zero to seven centrioles per cell (Figure 3D and Table S1). Other *Chlamydomonas* mutants with a similar variability in centriole number have been previously identified [25–27] and are referred to as *vfl* (variable flagellar number) mutants because the variable number of centrioles nucleates the assembly of variable numbers of flagella (Figure 3D) when the centrioles become basal bodies. These mutant phenotypes are thought to result from defective centriole segregation [28] and from defects in centriole mother–daughter cohesion [25,29].

The similarity between the variable flagellar number phenotypes of *asq2* and the *vfl* mutants raised the possibility that the *vfl* mutants might also share the centriole positioning phenotype. We therefore tested *vfl2* and *vfl3* for defects in centriole positioning and found when analyzed using our computational strategy that these mutants have centriole positioning defects comparable with those of *asq2*. *vfl2* cells have a mean θ_centriole_ of 55.2 ± 28.8° (Figure 3E, *n* = 64) and *vfl3* cells have a mean θ_centriole_ of 59.4 ± 35.2° (Figure 3F, *n* = 90). Genetic mapping studies show that *asq2* is not an allele of any of the previously described VFL genes (unpublished data).

### The Mother Centriole Instructs the Daughter Centriole to the Proper Subcellular Location

Using these mutants, we can begin to ask which component of the centrosome responds to polarity cues during positioning. The centrosome is composed of a mother centriole, a daughter centriole, and pericentriolar material, and is attached to the nucleus. In *Chlamydomonas,* these structures are spatially distinct but connected by fibers. Mother–daughter pairs are linked by striated fibers and connected to the nucleus by rhizoplasts [28,30] in *Chlamydomonas* and by Hook/Sun domain proteins in other organisms [31,32]. In principle, any of these components (the mother centriole, the daughter centriole, or the nucleus) could localize the others in response to polarity cues.

**Figure 4:**
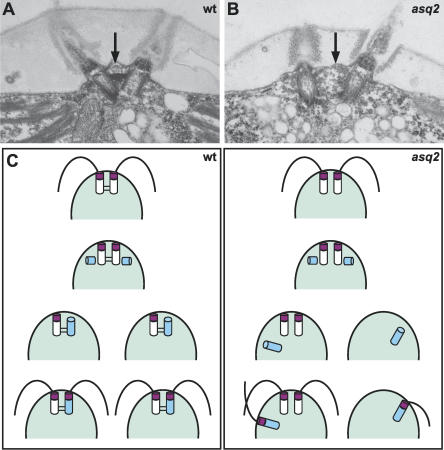
Using *asq2* Cells to Test the Role of the Mother Centriole (A) Electron micrograph showing electron-dense connecting fibers (distal striated fiber, denoted by arrow) joining mother and daughter centrioles. (B) Electron micrographs of *asq2* cell showing that centriole connecting fibers are missing. (C) Model for centriole positioning by mother centriole. In wt cells (left box), two centrioles are localized to the apical pole. These centrioles are connected by electron-dense connecting fibers (see [A], arrow). During duplication, each centriole will serve as a mother (white) to give rise to a daughter centriole (blue). New connections will form between each new mother–daughter pair. One mother–daughter centriole pair will be segregated to each cell following cell division. Each centriole will give rise to a flagellum, resulting in two cells with two centrioles and two flagella. In *asq2* cells (right box), centrioles are no longer connected (see [B], arrow). As in wt cells, each centriole will serve as a mother (white) to give rise to a daughter centriole (blue). However, because mother and daughter centrioles are no longer connected, centrioles will not segregate properly following mitosis, resulting in cells with variable numbers of centrioles. Among the centrioles that are distributed between cells, there will be a mix of mother and daughter centrioles. If the mother centrioles contain the necessary mark (purple) that allows them to find their proper subcellular location, whereas daughter centrioles are naive and unable to track to the correct place in the cell, then cells will have a population of properly positioned mother centrioles and a population of randomly localized daughter centrioles.

We first tested whether the mother centriole can localize the daughter or vice-versa. Previous studies have demonstrated that the *vfl* mutants result in dissociation of mothers from daughters and/or centrioles from the nucleus [28,29]. Using electron microscopy (EM), we verified that mother and daughter centrioles are likewise disconnected in *asq2* cells (Figure 4B). In wt cells, electron-dense fibers connect mother and daughter centrioles (Figure 4A, arrow). In contrast, *asq2* cells lack these connecting fibers (Figure 4B, arrow), confirming a loss of mother–daughter connections. These mutants therefore allow us to test which of these structures is able to localize properly when detached from the others.

Visual examination of *asq2* and *vfl* mutants suggested to us that the centriole distribution can be interpreted as a mixture of two populations: a population of correctly positioned centrioles (Figures 2E and S5B) and a population of randomly positioned centrioles (Figure 2D and 2E). On the basis of these observations and the known inherent disparity in maturation state between centrioles in each cell, we propose a model in which centriole maturity affects positioning. We considered a model in which the mother centriole is necessary for positioning the daughter centriole (Figure 4C). In accordance with this model, in the *asq1* class of mutants, the mother centriole can no longer respond to the cell polarity cue, and the mother–daughter pairs end up randomly localized. In the *asq2* class, the mother and daughter centrioles would be detached from each other, resulting in a population of properly positioned mother centrioles and a population of misplaced daughter centrioles. Because mother and daughter centrioles are no longer connected, centrioles will not segregate properly following mitosis, resulting in cells with variable numbers of centrioles. The key prediction of this model is that the mother centrioles in *asq2* cells should be properly localized, whereas the daughter centrioles should be improperly localized (Figure 4C).

**Figure 5:**
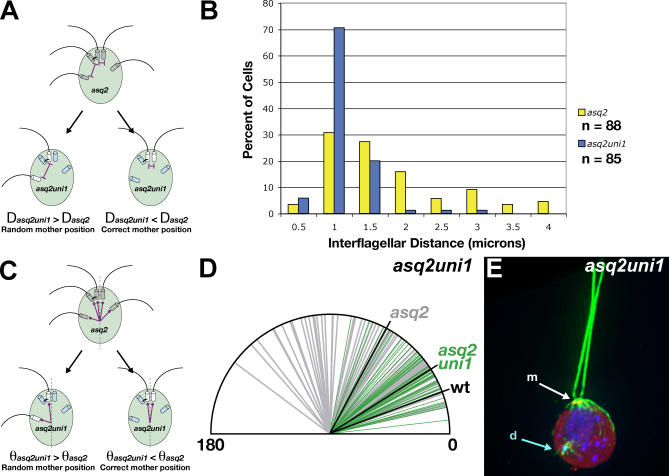
In *asq2* Cells, Mother Centrioles Are Properly Localized, Whereas Daughters Are Not (A) If mother centrioles (white) are properly positioned, then the distance between flagellated centrioles in *asq2uni1* mutant cells should be much smaller and less variable than that of single-mutant cells. (B) The distance between flagellated centrioles in biflagellate *asq2uni1* cells is much smaller and less variable (mean = 0.89 ± 0.36 μm, *n* = 85) than in biflagellate *asq2* cells (mean = 1.48 ± 0.85 μm, *n* =88). This difference is highly significant (one-tailed *t*-test, *p* < 1.23 e^−8^) (C) If mother centrioles (white) are properly localized, then the θ_centriole_ should be much smaller and less variable for flagellated centrioles in *asq2uni1* cells than for *asq2* cells. (D) θ_centriole_ for flagellated centrioles in *asq2uni1* cells is significantly (one-tailed *t*-test *p* < 2.02 e^−10^) smaller (green lines, mean θ_centriole_ = 32.4 ± 13.1°, *n* = 60) and less variable than in *asq2* cells (grey lines, mean θ_centriole_ = 61.7 ± 32.4°, *n* = 71). (E) Flagellated mother centrioles (m; white arrow) are properly localized in *asq2uni1* cells, whereas unflagellated daughter centrioles (d; blue arrow) are not. Cells are labeled with anti-acetylated tubulin and centrin antibody (green), anti-Bld10p antibody specific for centrioles (red) and DAPI (blue). Misplacement of nonflagellated daughter centrioles in *vfl2uni1* indicates that *uni1* does not simply suppress the centriole positioning phenotype of *vfl2*.

To test the prediction that mother centrioles are correctly positioned whereas daughters are mislocalized, we must be able to differentiate mother and daughter centrioles in 3D microscopy images. Mother centrioles have ultrastructural modifications that are lacking on daughter centrioles and are visible by EM, but serial section EM is not suitable for analyzing large numbers of cells. In order to be able to distinguish mothers and daughters in a more high-throughput manner, we employed a genetic strategy to render mother and daughter centrioles distinguishable by light microscopy. To do this, we took advantage of the *uni1* mutant in which flagella are formed predominantly by mother centrioles [33] (see flagellar distribution in Table S1). We then tested whether mother centrioles localize to the proper position at the apical pole by measuring the θ_centriole_ (Figure 1E) for all flagellated (mother) centrioles in *asq2uni1* double-mutant cells. If mother centrioles can respond to polarity cues, they should account for the properly positioned centrioles sometimes seen in *asq2* mutants, hence the mean θ_centriole_ of flagellated centrioles in *asq2uni1* cells should be smaller and less variable than that of *asq2* cells (Figure 5C). Indeed, we find that *asq2uni1* cells have a mean θ_centriole_ of 32.4 ± 13.1° (Figure 5D, green lines, *n* = 60), which is significantly (one-tailed *t*-test, *p* < 2.02 e^−10^) smaller than the mean θ_centriole_ for *asq2* cells (Figures 1H and 5D, grey lines). The mean θ_centriole_ for flagellated centrioles in *asq2uni1* cells is slightly higher than wt (Figure 1F, mean θ_centriole_ = 20.5 ± 9.0°) and *uni1* (Figure S2A, mean θ_centriole_ = 20.4 ± 8.5°), but this is expected because the *uni1* phenotype is incompletely penetrant, such that some daughter centrioles still bear flagella in *uni1* mutants (Table S1).

So as not to rely solely on the pyrenoid and cellular center of mass measurements, we employed an alternative measure of geometry based on distance measurements. We measured the 3D through-space distance between flagellated centrioles in *asq2uni1* cells. If mother centrioles localize to the same subcellular site, then the distance between flagellated centrioles should be relatively low in the double mutant, especially when compared to that of *asq2* cells in which both mother and daughter centrioles have flagella (Figure 5A, right). In contrast, if mother centrioles are randomly localized, then the interflagellar distance in *asq2uni1* double-mutant cells should be at least as large as in *asq2* cells and just as variable (Figure 5A, left). We find that in *asq2uni1* double mutants, the interflagellar distance is significantly smaller (Figure 5B, blue bars, mean = 0.89 μm ± 0.04 standard error of the mean [S.E.M.], *n* = 85) than that of *asq2* cells (Figure 5B, yellow bars, mean = 1.48 μm ± 0.09 S.E.M., *n* = 88) and less variable, confirming that mother centrioles cluster in the same subcellular location.

### The *uni1* Mutation Does Not Suppress the *asq2* Phenotype

An alternative explanation for these data is that the *uni1* mutation acts as a suppressor of the centriole segregation and/or positioning phenotype in *asq2* cells. Centriole number in *asq2uni1* cells (Figure S3A, mean centriole number = 1.67 ± 1.25, *n* = 317) is indistinguishable (one-tailed *t*-test *p* < 0.3) from that of *asq2* cells (Figure S4, *asq2* mean centriole number = 1.72 ± 1.27, *n* = 440), indicating that *uni1* does not suppress the centriole segregation defect.

Furthermore, *uni1* does not act as a suppressor of centriole positioning defects, because intercentriolar distance is similar in *asq2* (mean = 1.39 ± 0.94, *n* = 168) and *asq2uni1* (mean = 1.42 ± 1.12 , *n* = 174) cells (Figure S3B, one-tailed *t*-test, *p* > 0.39). The 3D immunofluorescence imaging of *asq2uni1* cells demonstrates that the mother and daughter centrioles remain detached in the double mutant just as in the *asq2* single mutant, demonstrating that the *uni1* mutation does not simply behave as a suppressor, either of the mother–daughter detachment phenotype or of the centriole mispositioning phenotype of the *asq2* mutation. Indeed, mother centrioles properly localize to the apical pole (Figure 5E, flagellated centrioles, white arrow), whereas disconnected daughter centrioles can wander to atypical sites (Figure 5E, unflagellated centriole, blue arrow). These observations confirm that mother centrioles are competent to be properly positioned and normally play an instructive role in leading the daughter centriole to the correct subcellular location. We therefore conclude that in *asq2* cells, centriole positioning is intact, because mothers can find the proper subcellular location, but daughters are mispositioned because they are detached from their mother.

### Centrioles Position the Nucleus

**Figure 6:**
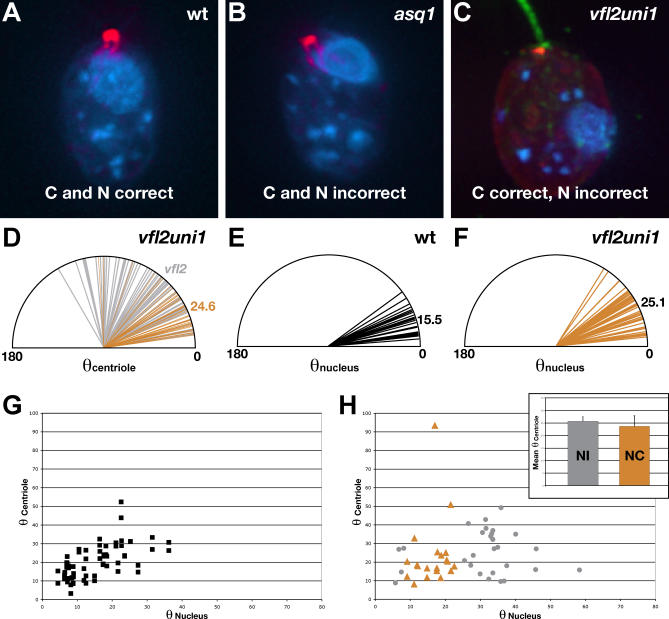
Centrioles Are Not Positioned by the Nucleus, but May Position the Nucleus (A) In wt cells, centrioles (red) are attached to the nucleus (blue) via centrin fibers (red). Both the centrioles (C) and nucleus (N) are properly localized near the apical part of the cell. Other plastid genomes are visible with DAPI staining (smaller blue dots). (B) *asq1* cell showing centrioles (red) and nucleus (blue) mislocalize together. (C) When centrioles are uncoupled from the nucleus in *vfl2uni1* cells, flagellated (green) mother centrioles (red) are properly localized to the apical side of the cell, whereas the nucleus (blue) can visit variable positions. (D) Mean θ_centriole_ for mother centrioles in *vfl2uni1* cells is 24.9 ± 14.7° (*n* =49, orange lines), which is significantly less than θ_centriole_ for *vfl2* cells (grey lines, one-tailed *t*-test *p* < 2.71 e^−11^), but not significantly different from wt. (E) wt cells have a mean θ_nucleus_ of 15.5 ± 8.1° (*n* = 58). θ_nucleus_ was determined by measuring the angle between the vector defining the long axis of the cell and a vector from the nuclear center of mass to the pyrenoid center of mass. (F) *vfl2uni1* cells have a significantly higher (one-tailed *t*-test, *p* < 2.9 e^−6^) mean θ_nucleus_ of 25.0 ± 11.8° (*n* = 49) compared to wt. (G) θ_nucleus_ and θ_centriole_ are correlated in wt cells, indicating that the position of the two organelles is coupled. (H) When the nucleus is detached from the centrioles in *vfl2uni1* cells, the nuclear position no longer correlates to centriolar position. Scatter plot visually shows loss of correlation between θ_centriole_ and θ_nucleus_. Points are color coded into two groups of cells, those with a nucleus whose position is within the correct wt range (defined as θ_nucleus_ less than one standard deviation from wt mean, and plotted in orange) and those with a nucleus whose position is incorrect (defined as θ_nucleus_ more than one standard deviation from wt mean, and plotted in gray). The two groups of points classified in this manner span the same range of values for θ_centriole_, further supporting a lack of correlation between nuclear and centriolar position when the nucleus is detached from the centriole. Inset: the mean θ_centriole_ (mean θ_centriole_ = 25.7 ± 11.3°, gray bar, *n* = 29) of cells with an improperly positioned nucleus (NI) is indistinguishable from the mean θ_centriole_ (mean θ_centriole_ = 23.7 ± 18.8°, orange bar, *n* = 20) of cells with a correctly positioned nucleus (NC). This shows that the mother centrioles can still attain the correct localization regardless of nuclear position.

Mother centrioles guide daughters to the correct subcellular position, but does the mother centriole play a role in instructing the position of other organelles? In a wt *Chlamydomonas* cell, the centrioles sit atop the nucleus and are attached to it by centrin-containing fibers called rhizoplasts [30] (Figure 6A). This juxtaposition suggests that centriole and nuclear positioning could be intimately linked. In most cell types, there tends to be a correlation between nuclear and centrosomal position. In *asq* mutant cells, the nucleus seems to be mispositioned along with the centrioles (Figure 6B), suggesting that centrioles position the nucleus or vice versa. A recent study has suggested that nuclear reorientation affects the position of the centrosome during cell migration in mammalian cells [9]. However, it has also been demonstrated that centrosomes are able to reach the cell cortex during *Drosophila* development without the aid of the nucleus [34]. To help address the controversy over who positions whom, nucleus or centrosome, we wanted to determine whether the nucleus could be impacting the localization of the mother centriole.

To test directly whether nuclear positioning has a causal impact on centriole position, we made use of the *vfl2* mutant in *Chlamydomonas* that has a mutation in centrin [35], a protein component of the rhizoplast. *vfl2* cells lack the centrin-based rhizoplast structure that connects the centrioles to the nucleus [28]. As shown in Figure 6D, *vfl2* centrioles have increased variability in positioning, but, like *asq2,* the mother centrioles remain properly localized at the apical pole as determined in *vfl2uni* mutants. We quantified nuclear position (θ_nucleus_) in *vfl2uni1, uni1,* and wt cells in a manner similar to the determination of θ_centriole_. We determined the long axis of the cell using the same method described above, but instead of marking each centriole, we obtained the nuclear center of mass and measured how much this point was shifted off the long axis of the cell. In wt cells, the mean angle θ_nucleus_ is 15.5 ± 8.1° (Figure 6E, *n* = 62). This value is similar to that of *uni1* cells (Figure S2B, θ_nucleus_ = 14.3 ± 5.6°, *n* = 40). In *vfl2uni1* cells, in which the nucleus has been uncoupled from the centrioles, the θ_nucleus_ is much more variable and the mean θ_nucleus_ (mean θ_nucleus_ = 25.0 ± 11.8°, Figure 6F, *n* = 49) is significantly higher (one-tailed *t*-test, *p* < 2.9 e^−6^), indicating that the nucleus is free to visit a wider range of positions once detached from the centrioles (Figure 6C). In contrast to the variable nuclear position, we find that, as in *asq2uni1,* in *vfl2uni1* cells, flagellated mother centrioles are properly localized, whereas the position of daughters is randomized (Figure 6D, *vfl2uni1* θ_centriole_ [orange lines], *vfl2* θ_centriole_ [grey lines]). *vfl2uni1* cells have a mean θ_centriole_ that is not statistically different (one-tailed *t*-test, *p* > 0.03 ) from wt or *uni1,* indicating that the mother centrioles can be correctly positioned despite the variable position of the nucleus.

We further tested whether the nucleus dictates centriole position, by measuring the correlation of nuclear position to that of centriole position on a cell-by-cell basis. In *vfl2uni* cells, θ_centriole_ for flagellated centrioles does not correlate with θ_nucleus_ (Figure 6H, *n* = 49, correlation coefficient of 0.10). When we compare the mean θ_centriole_ of cells with a correctly positioned nucleus (θ_nucleus_ is less that one standard deviation from the mean θ_nucleus_ for wt cells) to the mean θ_centriole_ of the cells with an incorrectly positioned nucleus (θ_nucleus_ is more than one standard deviation from the wt mean), the values do not differ significantly (one-tailed *t*-test, *p* > 0.33, Figure 6H, inset). These data indicate that the position of the nucleus has no obligatory impact on the position of centrioles in the cell and that correct centriole positioning in *Chlamydomonas* cells does not require attachment to the nucleus. Conversely, because the nucleus is mispositioned with the centrioles in *asq* mutant cells (Figure 6B), we wondered whether centrioles are involved in positioning the nucleus. In a population of wt cells, the θ_centriole_ correlates with θ_nucleus_ (correlation coefficient = 0.63, Figure 6G, *n* = 62). The fact that centriole position is unaltered and nuclear position randomized in a mutant that detaches centrioles from the nucleus, together with the fact that centriole position and nuclear position are correlated with each other when the centrioles are attached to the nucleus by the rhizoplast, suggests that centrioles dictate the position of the nucleus rather than vice versa.

Recent studies in migrating cell lines demonstrated that nuclear reorientation is important in positioning the centrosome towards the leading edge of the cell [9]. However, these studies only measured translational position of the centrosome and therefore cannot rule out a model in which rotation of the centrosome drives nuclear movement rather than vice versa. It would be interesting to repeat those experiments in cells lacking the nucleus–centrosome connections.

### Other Cellular Structures Are Misplaced with the Centrioles in *asq* Mutants

In addition to the nucleus, we also found that the rootlet microtubules (acetylated microtubule bundles involved in cleavage furrow placement in *Chlamydomonas* cells) are mispositioned along with centrioles in *asq* mutants. We found that rootlets were co-localized with centrioles in 27/27 cells (representative image shown in Figure S4B). Additionally, the contractile vacuoles are also mispositioned with centrioles in *asq* mutants (DIC image shown in Figure 3B and 3C, immunofluorescence images shown in Figure S4). To measure the position of the contractile vacuole, we fixed cells and incubated them with an antibody against FMG-1 (a flagellar membrane glycoprotein [36]) that binds to protein in the flagellar membrane as well as in other membrane-bound structures, including the contractile vacuoles (Figure S4C, inset). The distance between the contractile vacuole and the centrioles does not differ significantly between wt cells (mean = 0.52 ± 0.07 μm, Figure S4C) and cells in which centrioles are misplaced as in *asq1* (mean distance = 0.49 ± 0.07 μm, wt compared to *asq1*, *p* < 0.06, Figure S4D), *asq2* (mean distance = 0.49 ± 0.08 μm, wt compared to *asq2*, *p* < 0.04, Figure S4E), or *bld2* cells (mean distance = 0.53 ± 0.07 μm, *bld1* compared to *bld2*, *p* < 0.02, *bld2* compared to wt, *p* < 0.31, Figure S4F). We conclude that both rootlets and contractile vacuoles remain co-localized with centrioles even when centrioles are displaced, suggesting that centrioles may play a role in positioning these structures. Strictly speaking, because we do not have mutations that separate contractile vacuoles or rootlets from centrioles, we cannot definitively conclude whether the centrioles position these structures, or vice versa. However, we do note that in *asq2uni1* double mutants, rootlets can be seen associated with misplaced daughter centrioles in cells in which the mother centrioles have properly localized at the anterior pole (e.g., Figure 4E), suggesting that at least in this mutant, mother centrioles respond properly to the cell polarity cue, whereas the rootlets can be misplaced. The differential ability of the mother versus the daughter to respond to the polarity cue, despite no difference in their rootlet associations, tends to suggest that the mother, rather than the rootlets, is the primary responder to the polarity cue, although more complex models remain possible.

We also note that although the nucleus, rootlets, and contractile vacuole appear to co-localize with misplaced centrioles, this is not true of other structures, such as the pyrenoid or eyespot. The data therefore suggest that centrioles may influence the geometry of a specific subset of cellular structures, with other structures being independently oriented by a cell polarity system upstream of normal centriole positioning.

### The Distal Ends of Centrioles May Play a Role in Positioning

**Figure 7:**
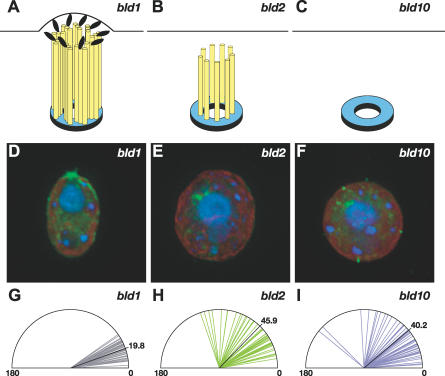
Mutant Centrioles with Defective Distal Ends Are Mispositioned (A) Centrioles contain nine triplet microtubule blades (yellow) arranged around a central cartwheel that sits on an amorphous disc structure (blue). At the most distal ends of centrioles in the region just proximal to the site of flagellar assembly, transition fibers are assembled (black ellipses) near the apical membrane. *bld1* mutant cells have normal centrioles and transition fibers, but are defective in flagellar assembly due to a loss of intraflagellar transport. (B) *bld2* cells are defective in centriole assembly and lack the B- and C-tubule of the triplet microtubule blades. As a result, the distal portion of *bld2* centrioles is missing. (C) *bld10* cells lack centriolar microtubules and have just the very proximal portion of the centriolar structure. (D) *bld1* centrioles (green represents centrin/acetylated tubulin labeling) localize to the apical membrane. (E and F) *bld2* and *bld10* cells have mispositioned centrioles (green) that appear in the cell interior. They are still found closely apposed to the nucleus (blue). (G) *bld1* cells have normally positioned centrioles (mean θ_centriole_ = 19.8 ± 8.0°, *n* =52) despite their lack of flagella. This demonstrates that neither flagella themselves, nor the intraflagellar transport machinery, is required for centriole positioning. (H) *bld2* centrioles lack the distal region and are mispositioned (mean θ_centriole_ = 45.9 ± 26.9°, *n* = 44). (I) *bld10* centrioles are also mispositioned (mean θ_centriole_ = 40.2 ± 30.8°, *n* = 46).

To begin to analyze which part of the mother centriole is responsible for positioning, we took advantage of known *Chlamydomonas* mutants with defects in centriole assembly, *bld2* and *bld10*. *bld2* cells have a mutation in epsilon tubulin [37] and are missing the B- and C-tubule of each of the nine triplet microtubule blades that normally comprise the centriole (compare Figure 7A and 7B). As a result, *bld2* centrioles have nine short, singlet microtubules and are lacking portions of the distal end. *bld10* cells, which are defective in the production of the centriole cartwheel-localized protein Bld10p, are missing all centriole microtubules and have at most just the most proximal portions of the centriolar structure [38].

Because *bld2* and *bld10* cells both lack flagella, we first determined the centriole positioning phenotype of *bld1* cells, which also lack flagella but have a structurally normal centriole. *bld1* cells have a mutation in the gene that encodes IFT52 [39]. These cells have centrioles that are structurally identical to wt cells, but due to a defect in a component of intraflagellar transport, they are unable to make flagella (Figure 7A). We found that *bld1* cells have a mean θ_centriole_ of 19.8 ± 8.0° (Figure 7G), similar to wt and demonstrating that assembly of flagella is not necessary for proper centriole positioning.

To determine whether the distal portion of the centriole is necessary for positioning, we measured the θ_centriole_ for *bld2* and *bld10* cells and compared it to θ_centriole_ for *bld1* cells. *bld2* cells have a mean θ_centriole_ of 45.9 ± 26.9° (Figure 7H), and *bld10* cells have a mean θ_centriole_ of 40.2 ± 30.8° (Figure 7I). These values differ significantly from those of *bld1* cells (*bld2:* one-tailed *t*-test, *p* < 5.4 e^−8^, *bld10:* one-tailed *t*-test, *p* < 3.1 e^−5^), which indicates that the distal portion of the centriole may be necessary for positioning. One potential explanation for the mispositioning of centrioles in *bld2* and *bld10* cells is that the centrioles are not actually attached to the cell surface. In many *bld2* and *bld10* cells (Figure 7E and 7F, respectively), centrioles appear in the cell interior and not at the apical membrane as in *bld1* cells (Figure 6D) and wt cells (Figure 2A). Therefore, structures at the distal ends of centrioles such as the transition fibers (Figure 7A) may be responsible for properly positioning the mother centriole by docking the centriole onto the cell surface.

## Discussion

### Towards a Pathway of Centriole Positioning

These data highlight a set of gene products required for proper centriole positioning (Table 1), which will serve as a starting point for a molecular dissection of the centriole positioning pathway. Moreover, the data support a model in which the mother centriole plays a role in establishing cell geometry. Particularly, the mother centriole leads the daughter to the proper location. Additionally, the centrioles position the nucleus and may position the rootlet microtubules and contractile vacuoles.

**Table 1:**
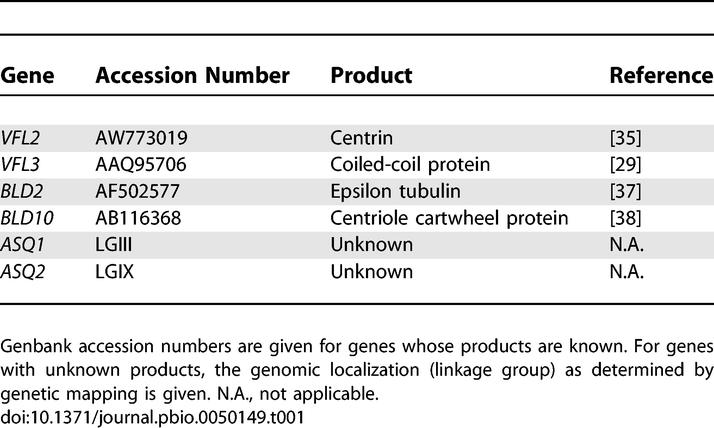
Genes Shown in This Study to Be Required for Centriole Positioning

Using the *uni1* mutation, we were able to distinguish between mature and immature centrioles in *asq2* and *vfl2* cells and determine their subcellular locations. One intriguing possibility is that at least some of the mispositioned unflagellated centrioles in *asq2uni1* and *vfl2uni1* cells are de novo–assembled centrioles, which are known to form in *vfl* mutants [40]. Because de novo–assembled centrioles are perhaps the most immature form of centrioles, this possibility would not invalidate our model that centriole maturity affects positioning. In fact, our model only presumes that mature centrioles can find their way to the proper subcellular site, whereas immature centrioles (which could include both templated daughter and de novo–assembled centrioles) cannot.

### Do Specialized Regions of Cortex Exist on Which Centrioles Can Dock?

An alternative model to explain centriole positioning is that there are only two slots for centrioles to dock into at the correct apical location, such that any cell with more than two centrioles would have more centrioles than could dock into these slots, and the extra centrioles would be mispositioned by default (an equivalent model for the case of ciliates was proposed [1]). Although cells with three or more centrioles per cell occur in *vfl2* and *asq2* populations (e.g., Figure S3A), those cells represent a small fraction of the population and hence would not account for the large increase in θ_centriole_ on average. Furthermore, a strong prediction of this model is that any cell with only one or two centrioles should have properly positioned centrioles because the two slots could accommodate these centrioles. However, we often observe cells with one or two centrioles that are clearly not at the correct position (Figures 2D and S5A), and conversely, we also see cells with more than two centrioles in which centrioles are clustered near the apical pole. Competition for a limited number of docking sites alone cannot explain these data. Therefore, although there may be specific docking sites on the cell surface, these sites alone are not sufficient to drive correct centriole positioning. There may in fact be a two-component system involving a specialized region at the cortex at which competent centrioles could dock.

Although we therefore do not think that saturation of a small, discrete set of docking sites can explain our data, our results are in no way inconsistent with the idea that a defined subregion of the cortex is set aside as a docking region. Indeed, just such a docking zone has been shown to exist in surf clam [41] and the marine worm *Chaetopterus* [42], in which it plays a key role in spindle attachment. A similar region exists in ascidians, known as the centrosome-attracting body, which plays a key role in asymmetric cell division during early embryogenesis [43]. The mother centriole could be interpreting a global polarity cue and tracking to a specialized cortical region, where it would be able to read out aspects of cell polarity to the position of other cellular structures. Alternatively, the mother centriole could itself be the mark to establish aspects of cell polarity. In Caenorhabditis elegans embryos, the paternally contributed centrosome is the early symmetry-breaking mark that induces a local change in the cortex and thereby establishes the anterior-posterior axis [44]. A similar role for centrioles in cell polarity is supported by the observation that *bld2* and *bld10* cells are often more round than are wt cells (compare cell shape in Figure 7E and 7F to Figure 2A), perhaps indicating a perturbation in global cell polarity. Because centrioles do not appear docked onto the cell surface in *bld2* and *bld10* cells, the centriole may require its distal portion not only for positioning, but also for exerting its effect on cell polarity. The mother centriole has structural appendages in the subdistal region that may couple centriole position and orientation with cell geometry through the cytoskeletal network.

### The Mother Centriole as a General Coordinator of Cell Geometry

A model in which the mother centriole can impact and propagate local cell geometry is appealing in light of experiments in ciliates [5,45,46] and vertebrate ciliated tissues [47] that demonstrate that ciliary orientation is dictated and propagated by a heritable local mark. These prior experiments demonstrated that a heritable mark exists, but were not able to reveal the identity of this mark because they could not dissociate the cellular components from one another. For instance in *Paramecium,* thousands of cilia are arranged into rows, with each cilium arising from a cortical unit. If rows of cilia are inverted from their normal orientation, the inverted orientation can propagate during cell division [5]. However, each cortical unit contains not only a cilium and centrioles, but also kinetodesmal fibers, trichocysts, striated bands, infraciliary lattice fibers, the “fork/bone node” [48], and an apparently self-duplicating oriented structure called the “post” [49]. Because inversion of rows simultaneously inverts the orientation of all of these other structures [50], it is not possible to determine which of the substructures within the cortical unit serves as a coordinating local signal to orient the other structures during formation of new cortical units in cell division.

The difficulty in interpreting the results of ciliate micromanipulation studies arises because such procedures leave the interactions between centrioles and other cortical structures intact, making it impossible to say who is positioning whom. In contrast, genetic manipulation using *Chlamydomonas* mutants allowed us to separate mother and daughter centrioles from each other and from other oriented structures, permitting us to determine that the local signal responsible for inheritance of orientation appears to be the mother centriole.

The differential potential of older versus more recently assembled structures has also been documented in higher organisms. Recent studies in *Drosophila* male germline [51] have shown that the mother centrosome behaves differently from the daughter centrosome during asymmetric cell division. Specifically, the mother centrosome is always inherited by the stem cell, whereas the daughter centrosome is inherited by the differentiating cell. The mother centriole may therefore be playing a similar role to the results described here in impacting aspects of cell geometry in metazoans. The fibrous connections between organelles have been intensively characterized in *Chlamydomonas,* but similar physical connections exist in vertebrate cells, for example between the mother and daughter centrioles and between centrioles and the nucleus [52,53], indicating that the mother centriole has the potential to coordinate cell geometry in a broad range of organisms. Although *Drosophila* can develop without centrioles [54], there is a clear requirement of centrioles in ciliated cells. Flies lacking centrioles are sterile and uncoordinated, indicating that sperm and potentially asymmetric cell divisions are perturbed. In this context, the role of centriole positioning may be in properly placing a cilium. Ciliary positioning is critical in higher vertebrates, for example in the establishment of left–right asymmetry [14] and in effective mucus clearing in the airway [15], where coordinated rotational orientation of the basal bodies is necessary to drive coherent flow of fluid across the epithelial surface. Abnormalities in cilia positioning due to defects in centriole migration have been observed in human patients [52], indicating that defects in centriole positioning may represent a specific class of ciliary disease. Because spindles can form in the absence of centrioles by a centrosome-independent pathway, there may be a similar fail-safe pathway for organizing other aspects of cell geometry.

The centriole is unique among cellular structures in its complexity, chirality, stability, and templated replication, and these features make it an ideal hub around which to organize and propagate particular aspects of cellular geometry. In particular, the fact that a mother centriole can not only produce a daughter, but instruct the daughter centriole concerning the correct positioning within the cell provides a potential basis for the phenomenon of “cytotaxis” [2] as the ability of a pre-existing cellular structure to determine the position or organization of newly formed cellular structure during cell replication. Our results have implications for the general problem of organelle positioning and cell geometry. The ability of the mother centriole to position the daughter and to orient the nucleus suggests that a complete understanding of organelle positioning will require analysis not only of individual organelles, but also of the pairwise mechanical linkages that may exist among distinct organelles.

## Materials and Methods

### Strains and culture conditions.

C. reinhardtii cells were grown and maintained in Tris-acetate-phosphate (TAP) media [55]. To generate insertional mutants to screen for phototaxis defects, the cell wall-less strain CC-849, *cw10* was electroporated [56] with linearized plasmid DNA containing the *aph7* gene, which confers resistance to hygromycin [57]. Strains were backcrossed to a wt strain of the opposite mating type (CC-125), and tetrads were dissected as previously described [55]. Double-mutant strains were constructed by crossing the pertinent single mutants and choosing spores from NPD tetrads that showed a non-wt phenotype.

### Immunofluorescence and microscopy.

Cells were fixed with Lugol's iodine solution to maintain robust cell geometry and prevent flagellar shearing and allowed to adhere to polylysine-coated coverslips. Cells were permeabilized with methanol and blocked with 5% BSA, 1% coldwater fish gelatin and 10% normal goat serum in PBS. Cells were then incubated in primary antibodies followed by secondary antibodies (Jackson ImmunoResearch, http://www.jacksonimmuno.com) diluted in 20% block, with six washes of 20% block in between. Cells were incubated with DAPI (diluted 1 μg/ml in water) and mounted in Vectashield mounting media on microscope slides. Slides were imaged using a 100× lens (numerical aperture [n.a.] = 1.4) on a Deltavision deconvolution microscope with an air condenser for DIC imaging. Images were processed and manipulated using Softworx image processing software.

### *asq* measurements.

Cells were fixed and stained as described above. For *asq* analysis, cells were labeled with DAPI and antibodies against centrin (diluted 1:100; a generous gift from J. Salisbury), acetylated tubulin (diluted 1:100; Sigma, http://www.sigmaaldrich.com), and Bld10p (diluted 1:100; a generous gift from M. Hirono), which together allow unambiguous identification of centrioles. A 3D stack through each cell was generated and used in the *asq* analysis. Using Softworx software, the center of mass of the nucleus, pyrenoid, and cell were defined. The center of mass was determined by obtaining the centroid, approximated by the midpoint of the three orthogonal edges of a bounding box containing the structure of interest and whose edges were parallel to the *x*-, *y*-, and *z*-axes of the 3D image. The appropriate structure for each specific θ measurement (e.g., the centrioles for θ_centriole_) were also marked. These coordinates were entered into a PERL script to calculate θ.

### Statistical analysis.

Comparison of means was performed using a one-tailed Student *t*-test in Excel. Unless indicated, error is shown as the standard deviation of the mean. For measuring correlation of datasets, the Pearson correlation coefficient was used.

## Supporting Information

Figure S1 Results from Cell Geometry Screen (A) Phototaxis was assayed using an opaque tube rack with a horizontal slit that permits light to strike the center of each test tube in the rack. When the door is closed (inset), light enters the rack only from through the slit. Light from a 25-W fluorescent bulb with an intensity of approximately 8,000 lux was used.
(B) Cells that phototax (ptx+) form a band at the level of a light source in about 10 min.
(C) Cells that are defective in phototaxis (ptx−) are uniformly present throughout the tube. Mutant lines that were defective in phototaxis were retained and re-screened by DIC microscopy to identify defects in cellular morphology.
(D) A total of 252 phototaxis-defective mutants are categorized into 15 phenotypic classes: askew *(asq),* no flagella *(bld),* uniflagellate *(uni),* stumpy flagella *(stumpy),* short flagella *(shf),* long flagella *(lf),* unequal length flagella within a cell *(ulf),* variable length flagella within population *(vlf),* clumpy groups of cells *(clumpy),* cell size, cell shape, cells look unhealthy *(sick),* defective eyespot *(eyespot),* other various phenotypes (other), and normal morphology *(norm).* Cells with variable flagellar numbers *(vfl)* are contained within the *asq* class of mutants.
(906 KB PDF)

Figure S2 Centriole and Nuclear Positioning in *uni1* Is Similar to wt (A) *uni1* cells have a mean θ_centriole_ of 20.4 ± 8.5° (*n* = 40), which is not statistically different from that of wt.
(B) *uni1* cells have a mean θ_nucleus_ of 14.3 ± 5.6° (*n* = 40).
(C) Centriole and nuclear position is highly correlated in *uni1* (correlation coefficient = 0.79)
(105 KB PDF)

Figure S3 The *uni1* Mutation Does Not Suppress Centriole Number or Position Defects in *asq2uni1* Cells (A) *asq2uni1* cells have a mean of 1.67 ± 1.25 (blue bars) centrioles per cell. This number is not statistically different (one-tailed *t*-test, *p* > 0.30) from that of *asq2* cells (yellow bars), which have a mean centriole number of 1.72 ± 1.27.
(B) Intercentriolar distance in *asq2* (mean = 1.39 ± 0.94, *n* = 168, yellow bars) cells is similar to that of *asq2uni1* cells (mean = 1.42 ± 1.12 , *n* = 174, blue bars, one-tailed *t*-test: *p* > 0.39)
(151 KB PDF)

Figure S4 Other Structures Are Misplaced in *asq* Cells In all panels, cells are oriented so that the pyrenoid is at the bottom of the cell. For rootlet visualization (A and B), cells were fixed and incubated with antibodies against acetylated tubulin (green) and Fla10 (red).
(A) In a wt cell, acetylated microtubule bundles emanate from near the centrioles. Normally, these rootlets are draped over the apical pole of the cell.
(B) When centrioles are misplaced in *asq* cells, the rootlet microtubules are also misplaced (27/27 cells), suggesting that either centrioles position the rootlet microtubules or vice versa.
(C–F) For contractile vacuole visualization, cells were fixed and incubated with antibodies against centrin (green), DAPI (blue), and FMG-1 (white), a protein that is present in the flagellar membrane as well as other membrane-bound structures [36]. FMG-1 signal alone is shown in the inset (C–F). Images represent single slices through 3D stacks of images.
(C) To measure positioning of the contractile vacuoles (CV) relative to the centrioles, the distance from each centriole (green) to each CV was measured. wt cells have a mean centriole to CV distance of 0.52 ± 0.07 μm (*n* = 39). Two CVs are visible at the apical side of the cell, as are other vesicular structures (potentially the Golgi) near the middle of the cell.
(D) *asq1* cells have a mean centriole to CV distance of 0.49 ± 0.07 μm (*n* = 37). Two CVs are visible.
(E) *asq2* cells have a mean centriole to CV distance of 0.49 ± 0.07 μm (*n* = 38). Three CVs are visible.
(F) *bld2* cells a mean centriole to CV distance of 0.53 ± 0.07 μm (*n* = 33). Two CVs are visible.
(2.3 MB PDF)

Figure S5 Single Centrioles Are Found in Correct and Incorrect Locations in *asq* Cells DIC (left panels) and fluorescence images (right panels) of *asq2* cells with one centriole. Cells are labeled with DAPI (blue) and antibodies against acetylated tubulin and centrin (green) and Bld10p (red). Images are oriented with the pyrenoid on the bottom.
(A) *asq2* cell with one incorrectly positioned centriole.
(B) *asq2* cell with one correctly positioned centriole.
(468 KB PDF)

Table S1 Distribution of Flagellar Number in Mutants (%) (37 KB DOC)

### Accession Numbers

The GenBank (http://www.ncbi.nlm.nih.gov/Genbank) accession numbers for the genes discussed in this paper are *BLD1* (AF397450), *BLD10* (AB116368), *BLD2* (AF502577), *VFL2* (AW773019), and *VFL3* (AAQ95706).

## Abbreviations

3D: three-dimensional
DIC: differential interference contrast
wt: wild-type
