# Supplementary material for: The Mother Centriole Plays an Instructive Role in Defining Cell Geometry
Source: PLoS Biol. 2007 May 22;5(6):e149. doi: 10.1371/journal.pbio.0050149 (PMC1872036; doi:10.1371/journal.pbio.0050149)

**Supplemental Figure S4.**

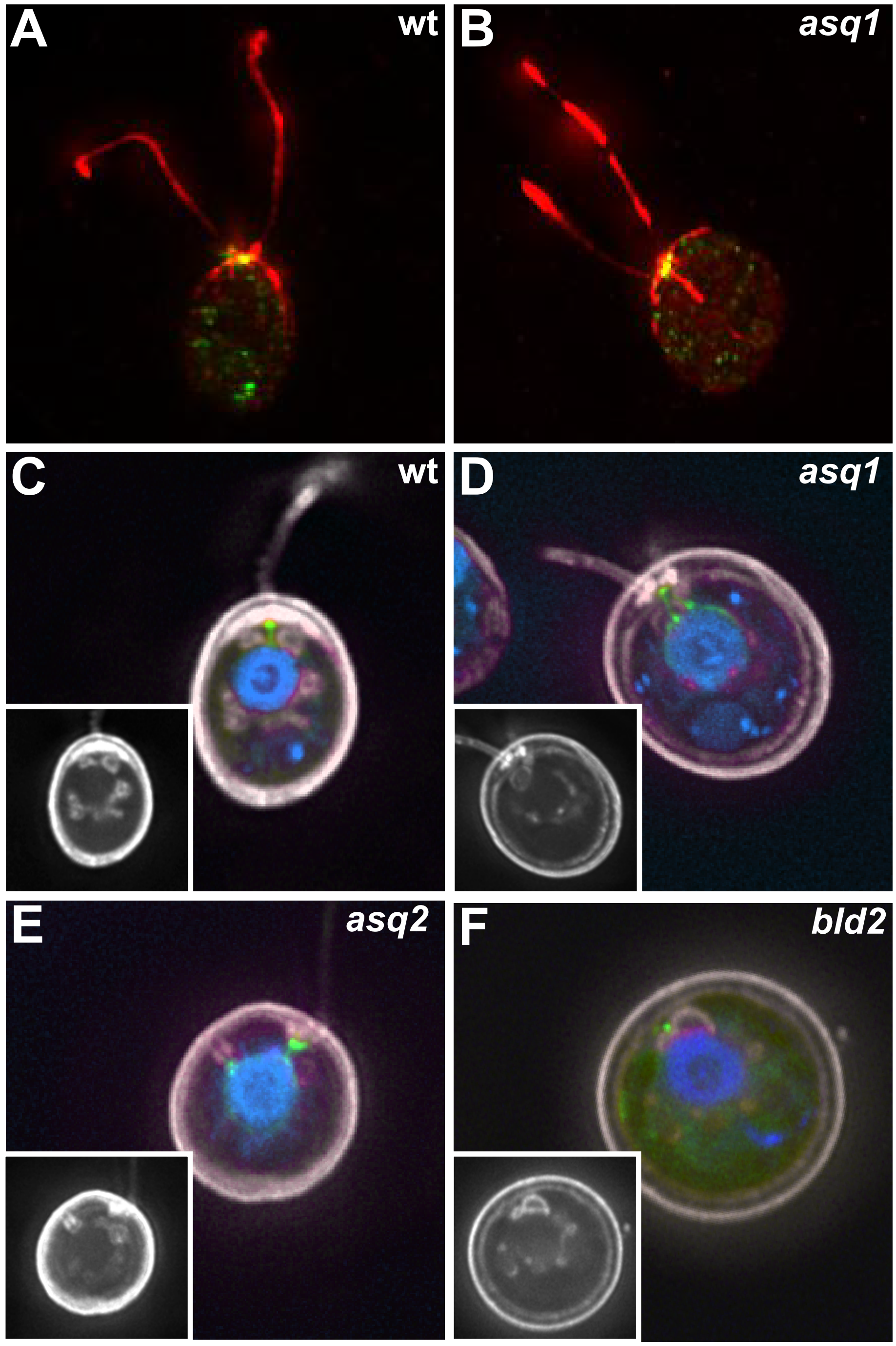

Supplement: Supplementary file 4 [file pbio.0050149.sg004.pdf]
