# Supplementary material for: The Mother Centriole Plays an Instructive Role in Defining Cell Geometry
Source: PLoS Biol. 2007 May 22;5(6):e149. doi: 10.1371/journal.pbio.0050149 (PMC1872036; doi:10.1371/journal.pbio.0050149)
Supplement: Supplementary file 6 [file pbio.0050149.st001.doc]

Supplemental Table 1. Distribution of Flagellar Number in Mutants (%)

|  | *n* | *0* | *1* | *2* | *3* | *4* | *5* | *6* | *7* |
| --- | --- | --- | --- | --- | --- | --- | --- | --- | --- |
| wt | 1005 | 2.8 | 0.6 | 96.6 | 0 | 0 | 0 | 0 | 0 |
| *uni1* | 176 | 34.7 | 58.5 | 6.8 | 0 | 0 | 0 | 0 | 0 |
| *asq2* | 1274 | 23.9 | 26.1 | 34.5 | 11.9 | 3.2 | 0.4 | 0 | 0.08 |
| *asq2uni1* | 290 | 36.9 | 37.9 | 22.4 | 2.8 | 0 | 0 | 0 | 0 |
| *vfl2* | 977 | 42.8 | 14.6 | 33.8 | 8.0 | 0.8 | 0 | 0 | 0 |
| *vfl2uni1* | 966 | 74.4 | 22.0 | 3.4 | 0.1 | 0 | 0 | 0 | 0 |
| *vfl3* | 466 | 45.5 | 11.6 | 32.4 | 7.3 | 2.6 | 0.7 | 0 | 0 |
| *vfl3uni1* | 607 | 59.6 | 29.8 | 9.7 | 0.8 | 0 | 0 | 0 | 0 |
